# Effectiveness of community interventions for protecting and promoting the mental health of working-age adults experiencing financial uncertainty: a systematic review

**DOI:** 10.1136/jech-2020-215574

**Published:** 2021-04-30

**Authors:** Michael McGrath, Fiona Duncan, Kate Dotsikas, Cleo Baskin, Liam Crosby, Shamini Gnani, Rachael Maree Hunter, Eileen Kaner, James Bowes Kirkbride, Louise Lafortune, Caroline Lee, Emily Oliver, David P Osborn, Kate R Walters, Jennifer Dykxhoorn, School for Public Health Research Public Mental Health Programme

**Affiliations:** 1 Division of Psychiatry, University College London, London, UK; 2 Department of Health Services Research and Policy, London School of Hygiene & Tropical Medicine, London, UK; 3 Department of Sport and Exercise Sciences, Durham University, Durham, UK; 4 Department of Primary Care and Public Health, Imperial College London, London, UK; 5 Department of Primary Care and Population Health, University College London, London, UK; 6 Institute of Health and Society, Newcastle University, Newcastle, UK; 7 Cambridge Public Health, University of Cambridge, Cambridge, UK; 8 Cambridge Institute for Sustainability Leadership, Cambridge, UK; 9 Camden and Islington NHS Foundation Trust, London, UK

**Keywords:** inequalities, mental health, psychosocial factors, public health, systematic reviews

## Abstract

**Background:**

The COVID-19 pandemic has created a period of global economic uncertainty. Financial strain, personal debt, recent job loss and housing insecurity are important risk factors for the mental health of working-age adults. Community interventions have the potential to attenuate the mental health impact of these stressors. We examined the effectiveness of community interventions for protecting and promoting the mental health of working-age adults in high-income countries during periods of financial insecurity.

**Methods:**

Eight electronic databases were systematically screened for experimental and observational studies published since 2000 measuring the effectiveness of community interventions on mental health outcomes. We included any non-clinical intervention that aimed to address the financial, employment, food or housing insecurity of participants. A review protocol was registered on the PROSPERO database (CRD42019156364) and results are reported in accordance with Preferred Reporting Items for Systematic Reviews and Meta-Analyses guidelines.

**Results:**

From 2326 studies screened, 15 met our inclusion criteria. Five categories of community intervention were identified: advice services colocated in healthcare settings; link worker social prescribing; telephone debt advice; food insecurity interventions; and active labour market programmes. In general, the evidence for effective and cost-effective community interventions delivered to individuals experiencing financial insecurity was lacking. From the small number of studies without a high risk of bias, there was some evidence that financial insecurity and associated mental health problems were amenable to change and differences by subpopulations were observed.

**Conclusion:**

There is a need for well-controlled studies and trials to better understand effective ingredients and to identify those interventions warranting wider implementation.

## Introduction

The COVID-19 pandemic has created an unanticipated period of global financial insecurity.^1^ The introduction of measures to contain the virus and protect health systems has resulted in increased unemployment and job insecurity,^2^ personal debt,^3^ food insecurity^4^ and mortgage and rent stress,^3 5^ all of which are risk factors for poor mental health.^6–10^ Financial strain, indebtedness and the inability to provide materially for one’s family may increase stress and household conflict, undermine personal autonomy and induce feelings of shame and guilt.^11–13^ Further, the transition out of secure housing and employment can disrupt structures that offer emotional stability, reduce social connectedness and remove the sense of purpose and fulfilment that employment can provide.^11–14^ As a result, periods of financial uncertainty are associated with the incidence of common mental disorders,^15 16^ increased symptoms of severe mental illness^17^ and suicide and self-harm.^18^

These relationships are bidirectional, reflecting both social causation and social drift.^19^ Adverse financial events increase the risk of developing mental illness (ie, social causation),^14 20^ and poor mental health can exacerbate financial insecurity due to experiences of stigma and discrimination, difficulties finding and maintaining employment and strained family and personal relationships (ie, social drift).^14 21^ Further, differentials in exposure and vulnerability across the socioeconomic gradient mean these stressors are concentrated in deprived communities and have the potential to widen inequalities.^22^ Longitudinal analysis of poverty dynamics in high-income countries shows that compared with a relatively small number of people experiencing sustained poverty, there is a substantially larger population moving in and out of poverty from 1 year to the next.^23^ Importantly, longer episodes of poverty are associated with a reduced likelihood of exiting poverty and a higher risk of re-entering poverty in the future.^24^ Therefore, periods of financial adversity represent a crucial juncture for preventing common mental disorders, promoting mental well-being and reducing social inequalities.

Community interventions present one means of attenuating the mental health impact of episodes of financial insecurity.^25^ These interventions draw on resources and expertise within communities and beyond the healthcare system to provide individuals with non-clinical forms of support to improve their psychosocial circumstances.^26^ By acknowledging that individuals are embedded within wider social, economic and political contexts, community interventions operate across all levels of the socioecological model of health promotion (from the individual to the policy level) to prioritise both health and social outcomes.^26–29^

While implementing interventions that act on the social determinants of mental health has been highlighted as a global priority, a substantial implementation gap exists.^30 31^ Barriers to wider implementation include limited evidence of effectiveness, the complexity of addressing social and economic challenges, and that action can be seen as inherently political.^32^ As the economic fallout of the COVID-19 pandemic unfolds, there have been renewed calls to pre-empt the mental health impacts of increasing household debt, unemployment and food insecurity by implementing interventions that provide social and financial support.^33 34^

Previous reviews have examined the effectiveness of community interventions for improving the mental health of specific subpopulations, such as the long-term unemployed,^35 36^ people experiencing homelessness^37 38^ and people with pre-existing mental illness.^39 40^ However, a stronger evidence base regarding the delivery of these interventions to the general population is required. A review of randomised controlled trials (RCT) of interventions aiming to reduce the impact of financial hardship on mental health found limited evidence of effectiveness for most types of interventions.^41^ All participants in the studies identified were unemployed, and the authors emphasised the need for further research and evaluation of interventions delivered in both primary care settings and within statutory and voluntary sector organisations. Therefore, we examine community interventions that seek to address acute financial stressors and their consequences (such as recent or imminent job loss, debt and legal issues, and housing and food insecurity) in a broader population and draw on evidence from a wider range of study designs. Specifically, this systematic review aims to (1) determine the effectiveness of community interventions for improving the mental health of working-age adults in high-income countries during periods of financial uncertainty, and (2) evaluate the impact of these interventions on health inequalities.

## Methods

We conducted a systematic review to identify effective community interventions for improved mental health and highlight priorities for future research. A review protocol was registered on the PROSPERO database (CRD 42019156364) and results are reported in accordance with Preferred Reporting Items for Systematic Reviews and Meta-Analyses guidelines.^42^

### Search strategy and screening

An electronic search of eight academic and grey literature databases (Embase, Medline, PsycINFO, Web of Science, CINAHL Plus, the Cochrane Library, OpenGrey, Social Care Online) was conducted in August 2019. The search strategy incorporated a mix of keywords, and subject headings adapted for each database. Based on our study aims, the search strategy was built around three concepts: (1) personal or household financial insecurity, including recent unemployment, precarious employment, personal debt, food and housing insecurity; (2) community interventions; and (3) mental health and well-being outcomes (see online supplemental material). Two reviewers independently screened titles, abstracts and full-text articles, with discrepancies reconciled through discussion. Only studies reporting primary research and published in English since January 2000 were included. Additional studies were identified by manually searching relevant systematic reviews and study protocols identified during screening and backward-forward citation searching all studies meeting our inclusion criteria.

10.1136/jech-2020-215574.supp1 Supplementary data

### Inclusion and exclusion criteria

For inclusion, studies needed to report the effectiveness of a community intervention on mental health outcomes for working-age adults (aged 18–64) in high-income countries, as defined by the Organisation for Economic Co-operation and Development,^43^ experiencing personal or household financial uncertainty (table 1). We draw on an established definition of community interventions and include non-clinical programmes, services and policies that (1) operate at any level of health promotion (from the individual level to the policy level), (2) draw on resources and expertise found within communities and beyond the healthcare system, and (3) address both health and social outcomes.^26 28^ We included interventions adopting universal or selective prevention strategies, which attempt to attenuate the impact of financial insecurity on mental health and well-being in the general population, rather than in people with existing mental health problems.^44^ Interventions were included if they addressed acute episodes of financial insecurity and their consequences, such as programmes for the recently unemployed (defined as a period of less than 12 months) or those at risk of homelessness, the provision of legal or debt advice, or services for the food insecure, rather than those addressing persistent problems such as long-term unemployment or homelessness.

**Table 1:** Inclusion and exclusion criteria

Population	
Include	Working-age adults (18–64 years) living in high-income, OECD countries experiencing periods of personal or household financial uncertainty relating to employment (eg, recent or imminent unemployment, precarious employment), personal debt and legal issues, housing security (eg, mortgage or rent stress, threatened eviction) or food insecurity.
Exclude	Other chronic stressors or populations with more complex needs (eg, long-term homelessness, refugees and asylum seekers, recently released offenders, people experiencing domestic violence).
**Intervention**	
Include	Community or social interventions. Defined as any non-clinical programme, service or policy that draws on resources beyond the healthcare system to improve the psychosocial living conditions of participants or their community.
Exclude	Clinical or pharmaceutical interventions, including psychotherapies.
**Comparator**	
Include	Experimental or quantitative observational studies that employ either preintervention and postintervention measurements, or intervention and control arms.
Exclude	Studies without preintervention and postintervention measurements or a comparison group, formative and process evaluations, qualitative studies.
**Outcomes**	
Primary outcomes	Mental health outcomes broadly defined to include any objective measure of: Psychological distress (eg, worry, stress, shame). The symptoms of common mental disorders (eg, anxiety, depression). Well-being and positive affect (eg, quality of life, happiness, self-esteem, resilience). Mental health service utilisation (eg, consultations, referrals, prescribing).
Secondary outcomes	Any data relevant to the cost-effectiveness of the intervention (from an individual, societal or government perspective).

OECD, Organisation for Economic Co-operation and Development.

The primary outcome, mental health and well-being, was broadly defined to include psychological distress, the symptoms of common mental disorders, aspects of well-being and positive affect, and mental health service utilisation. In order to measure effectiveness, only quantitative studies employing experimental, quasiexperimental or observational designs (with either a non-exposed comparison group or before-after intervention measurement) were included. Data relevant to an economic evaluation were extracted as secondary outcomes.

### Data extraction and analysis

A data extraction framework was adapted from the Template for Intervention Description and Replication checklist^45^ and included the following elements: study location, participant characteristics, intervention description, financial stressor targeted, procedure and activities, mode/location of delivery, duration, frequency/intensity of intervention, study design, sample size, follow-up period and primary and secondary outcomes.

Two reviewers independently assessed individual study quality and risk of bias using the Effective Public Health Practice Project’s Quality Assessment Tool for Quantitative Studies.^46^ Mental health outcomes of each study were assessed on a three-point scale and against eight domains: selection bias, study design, confounders, blinding, data collection methods, withdrawals, intervention integrity and analysis. Discrepancies were resolved through discussion, and each study was given an overall quality rating of ‘high’, ‘moderate’ or ‘low’, following instrument scoring guidelines. Studies were not excluded based on their quality assessment.

## Results

**Figure 1:**
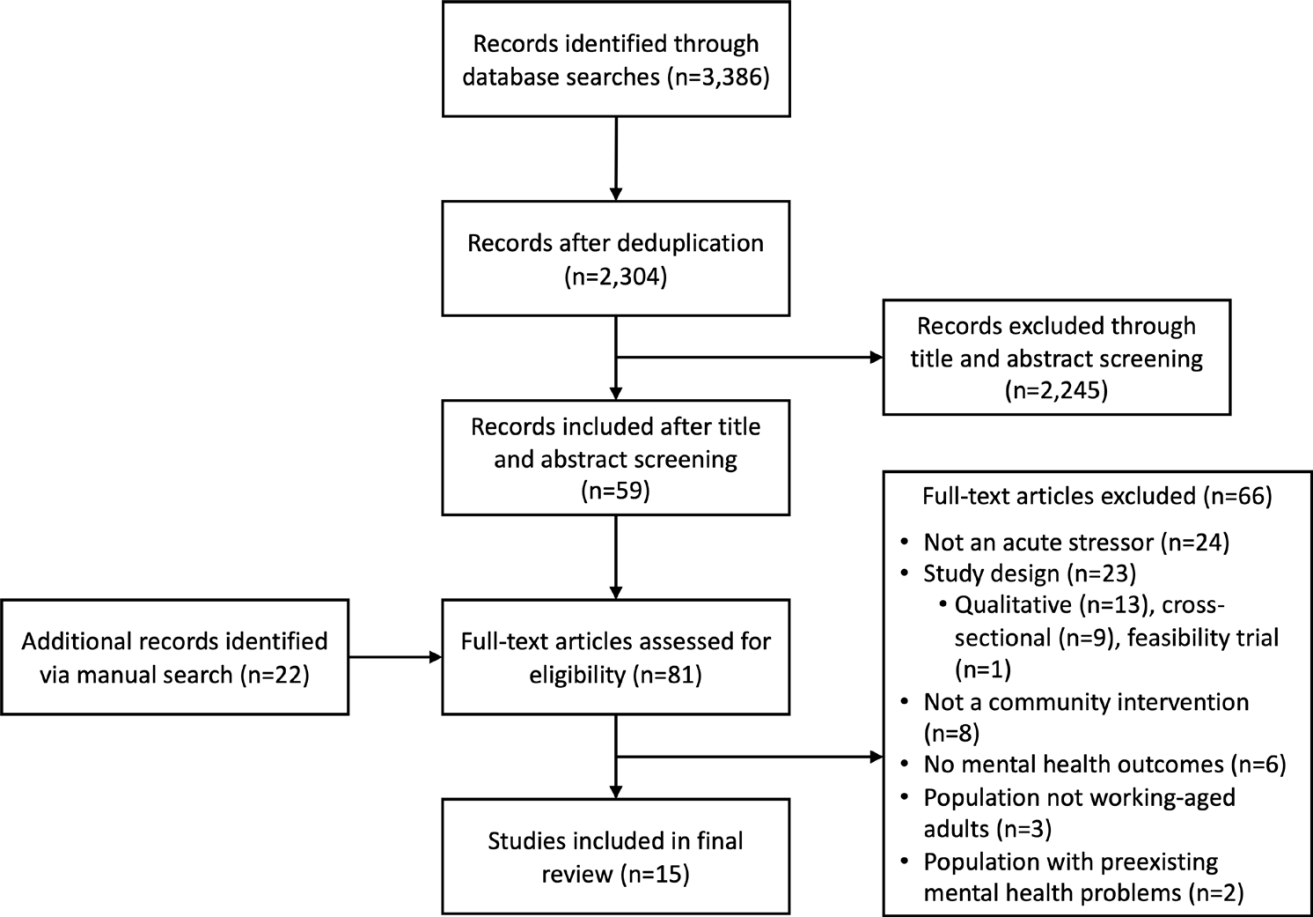
Preferred Reporting Items for Systematic Reviews and Meta-Analyses (PRISMA) flow diagram of included studies.

2326 non-duplicate titles identified were identified, 2304 from database searches and 22 from manual searches. Of these, 15 studies met our inclusion criteria (figure 1). Ten studies (67%) were identified from the UK, two (13%) from the USA and one study each (7%) from Finland, Germany and Canada. Four studies used experimental research designs (three RCTs^47–49^ and one quasiexperimental controlled study^50^) and the remaining 11 employed either controlled or uncontrolled before-after methods. Based on our quality assessment, three studies were rated ‘moderate’ quality^47 48 50^ and the remaining 12 studies ‘low’ quality.

Most studies (n=12) measured mental health outcomes using standardised screening instruments for the symptoms of anxiety and depression, stress, psychological distress, well-being and functioning (table 2). Four studies reported mental health service utilisation, including primary care consultations, referrals, and antidepressant, hypnotic and anxiolytic prescribing.^47 50–52^

**Table 2:** Summary of included studies

Study (year)	Participants and setting	Study design	Outcomes
*Welfare and advice services located in healthcare settings*			
Abbott and Hobby (2000)^53^	Service users (n=68, 60% female) in five general practices in a deprived area of Liverpool, England.	Uncontrolled before-after study (6 and 12-month follow-up)	SF-36
Caiels and Thurston (2005)^56^	Participants (n=96, 64% female, 98% White) in an advice service delivered at general practices in England.	Uncontrolled before-after study (follow-up at case closure)	SF-12
Abbott *et al* (2006)^54^	Welfare benefits advice delivered to service users (n=345, 53% female, 96% White British) recruited from 59 GP surgeries in England.	Uncontrolled before-after study (6 and 12-month follow-up)	SF-36
Ryan *et al* (2012)^55^	A medical–legal partnership for low-income earning patients (n=204, 72% female, 53% White) in a family clinic in Arizona, USA.	Uncontrolled before-after study (follow-up at case closure, mean: 2 months)	PSS-10, MYCaW
Harris (2013)^57^	Participants in an advice service delivered in a hospital in London (n=745, 66% female, 51% White).	Uncontrolled before-after study (follow-up at case closure)	SF-36, health service costs
Krska *et al* (2013)^52^	Patients (n=148, 65% female, 78% working age) at six general practices in deprived areas of England participating in an outreach service.	Uncontrolled before-after study (6-month follow-up)	Health service utilisation
Woodhead *et al* (2017)^50^	Adults (n=910, 63% female, 49% White British) accessing colocated welfare advice in eight general practices in London.	Quasiexperimental controlled study (3-month follow-up)	GHQ-12, SWEMWBS, health service utilisation
*Link worker social prescribing*			
Grant *et al* (2000)^47^	Patients (n=161, 75% female) in 26 general practices in England identified by their general practitioners (GPs) as having psychosocial problems and referred to local and national voluntary organisations.	Randomised controlled trial (1 and 4-month follow-up)	HADS, Duke-UNC FSSQ, health service utilisation
Grayer *et al* (2008)^51^	Link workers direct patients (n=108, 62% female, mean age: 43, 67% White) identified as having psychosocial problems in 13 general practices in London to community and voluntary services.	Uncontrolled before-after study (3-month follow-up)	GHQ-12, CORE-OM, health service utilisation
*Telephone debt advice services*			
Jinhee Kim *et al* (2013)^58^	Clients (n=355, 68% female, 65% White) drawn from the database of a non-profit credit counselling agency in the USA.	Controlled before-after study (18-month follow-up)	Composite health score, including experiences of stress
Pleasence and Balmer (2007)^48^	People (n=402) experiencing problems with debt recruited from 16 unemployment and welfare centres in England and Wales.	Randomised controlled trial (5-month follow-up)	STAI-S, EQ-5D
*Food insecurity interventions*			
Roncarolo *et al* (2016)^61^	Food insecure individuals (n=824, 58% female, 36% foreign born) recruited from 24 non-profit organisations in Montreal, Canada.	Controlled before-after study (9-month follow-up)	SF-12
*Active labour market programmes*			
Vinokur *et al* (2000)^49^	Recently unemployed people (55% female, median age: 35, 76% White, mean period of unemployment: 4.1 weeks) in Michigan, USA, participating in a week-long, group job search workshop.	Randomised controlled trial (2, 6 and 24-month follow-up)	HSCL, UM-CIDI, role and emotional functioning
Saloniemi *et al* (2014)^60^	Recently unemployed people (n=342, 53% female, mean age: 36, 71% unemployed for <12 months) in Finland participating in government-run vocational training courses.	Uncontrolled before-after study. Follow-up at programme conclusion (range: 3–24 months)	GHQ-12, SSS, self-reported sense of coherence
Rose (2019)^59^	9% random sample of all people aged 16–54 entering unemployment in Germany in 1 year.	Controlled before-after study (12-month follow-up)	Self-reported life satisfaction

CORE-OM, Core Outcome Measurement tools; Duke-UNC FSSQ, Functional Social Support Questionnaire; EQ-5D, EuroQol-5 Dimension; GHQ-12, General Health Questionnaire; HADS, Hospital Anxiety and Depression Scale; HSCL, Hopkins Symptoms Checklist; MYCaW, Measure Yourself Concerns and Well-being; PSS-10, Perceived Stress Scale; rOR, Ratio of odds ratios; SF-12, 12-item Short Form Survey; SF-36, 36-item Short Form Survey; SSS, Stress Symptom Scale; STAI-S, State-Trait Anxiety Inventory-state short form; SWEMWBS, Shortened Warwick-Edinburgh Mental Well-Being Scale; UM-CIDI, Composite International Diagnostic Interview, University of Michigan short version.

### Community interventions

Following data extraction, the included studies were categorised by the two reviewers into five intervention types: welfare and advice services colocated in healthcare settings (n=7),^50 52–57^ link worker social prescribing (n=2),^47 51^ telephone debt advice (n=2),^48 58^ active labour market programmes for the recently unemployed (n=3)^49 59 60^ and food insecurity interventions (n=1)^61^ (table 3).

**Table 3:** Types of community interventions identified

Type of intervention	Description
Welfare and advice services colocated in healthcare settings (n=7)^50 52–57^	Services operating in primary care and hospital settings that provide information, support and advocacy for people dealing with financial insecurity. These services assist participants with problems relating to welfare (eg, benefits entitlement and appeals), housing (eg, tenancy agreements, repossessions and eviction), employment problems (eg, redundancy, unfair dismissal and training), finances (eg, income maximisation and financial literacy) and legal issues (eg, debt, compensation and court action).
Link worker social prescribing (n=2)^47 51^	Trained link workers refer patients who present to their general practitioner with psychosocial problems to voluntary and community sector services, including cultural activities, befriending services and physical activity groups.
Telephone debt advice services (n=2)^48 58^	Advice delivered via telephone providing financial planning and budgeting, credit counselling, assistance with repossession and bailiffs, and referral services, in conjunction with self-help material delivered online or by post.
Active labour market programmes (n=3)^49 59 60^	Programmes delivered as part of national labour policies for the recently unemployed. These policies differ by country but involve early intervention when people become unemployed and regular monitoring of activities based on principles of ‘mutual obligation’. Unemployed people may be required to undertake job-seeking activities, participate in vocational and life skills training, complete work placements or volunteer their time in order to receive welfare benefits and avoid sanctions.^83^
Food insecurity interventions (n=1)^61^	Programmes that aim to alleviate food insecurity, including food banks, collective kitchens and bulk-buying clubs.

#### Welfare and advice service colocated in healthcare settings

Seven studies measured the effectiveness of colocated welfare and advice services on mental health outcomes. A quasiexperimental controlled study in London rated of moderate quality reduced self-reported financial strain among participants at 3-month follow-up, compared with controls.^50^ Overall, participants showed no changes in well-being, the symptoms of common mental disorders or self-reported general practice (GP) consultations. However, well-being scores did improve for those participants for whom advice resulted in positive financial outcomes, while symptoms of common mental disorders declined for women and Black participants.

A further six uncontrolled before-after studies, which were assessed to be of low quality, showed inconsistent results.^52–57^ Reduced stress and increased well-being were observed among low-income earning, legal advice recipients in a family practice in Arizona, USA.^55^ Three studies in England reported improvements in some, but not all, 36-item Short Form Survey domains for welfare and financial advice recipients.^53 54 57^ However, there was little evidence these improvements were sustained in the longer term. Another advice outreach service in deprived areas of England reported reductions in GP appointments and prescriptions for hypnotics and anxiolytics but found no evidence of a change in antidepressant prescribing.^52^

#### Link worker social prescribing

Two studies from the UK measured the effectiveness of link workers providing referral from primary care to voluntary and community sector organisations. Both studies focused on the mechanism of referral rather than the specific social prescription that patients received. An RCT in England used trained project facilitators to encourage participation in programmes managed by local and national voluntary organisations.^47^ The study failed to report all quality assessment domains adequately and received a moderate quality assessment. Compared with controls, at 4-month follow-up, participants in the intervention arm showed greater improvements in anxiety and functional health status. No difference was observed in depression or perceived social support. These patients also received more mental health prescriptions and fewer mental health referrals than controls. In an uncontrolled before-after study in London rated low quality, link workers directed, and if necessary, accompanied GP-referred patients to community programmes.^51^ At 3-month follow-up participants showed reductions in psychological distress, primary care consultations or mental health prescriptions.

#### Telephone debt advice services

Two studies describing telephone debt advice services were identified, and both received a low-quality rating.^48 62^ An RCT in England and Wales recruited individuals experiencing debt-related stress from unemployment centres.^48^ Participants in the intervention arm received telephone advice, which included assistance with repossession and bailiffs, financial planning, written self-help material and a referral service. The trial encountered difficulties recruiting participants and delivering telephone advice and, because of higher than anticipated attrition, stopped before completing 12-month follow-up. However, at 20 weeks, there was no evidence of improved anxiety or general health scores. A not-for-profit debt management programme in the USA provided telephone credit counselling and online material.^58^ In a before-after evaluation of the programme, at 18-month follow-up participants reported small improvements in a composite health score, which included self-reported stress.

#### Food insecurity interventions

A non-randomised study of food insecure, low-income households in Canada was rated low quality and compared traditional food banks to ‘alternative’ food insecurity interventions (collective kitchens, bulk-buying clubs and community gardens), which aim to promote empowerment, social inclusion and skill development.^61^ Improvements in food security and mental health were observed in food bank users at 9-month follow-up, but not for participants of alternative interventions.

#### Active labour market programmes for the recently unemployed

Three studies examined mental health outcomes following participation in active labour market programmes.^49 59 60^ An RCT in the USA assigned recently unemployed people into either week-long, job search workshops or a control group receiving an advice booklet.^49^ The intervention comprised daily 4-hour group sessions, which aimed to increase participants’ sense of mastery and motivation by teaching job search skills and protecting against setbacks. At 2-year follow-up, participants in the intervention arm had lower depression scores, higher levels of role and emotional functioning, and were less likely to have experienced a major depressive episode in the previous year. The study received a moderate rating as not all quality assessment domains were reported.

Another two programmes for the recently unemployed were identified and received low-quality assessments. In Germany, participants in labour schemes who received wage and self-employment subsidies or undertook training courses showed increased life satisfaction at 12-month follow-up, compared with baseline (measured within 2 weeks of becoming unemployed).^59^ At the conclusion of vocational training programmes in Finland, no improvements were observed in participants’ psychological distress, stress symptom scores or sense of coherence, compared with baseline.^60^ Nevertheless, subgroup analyses revealed improvements in these outcomes for people with a tertiary education, those studying courses in ‘white-collar’ occupations, but not for those studying ‘blue-collar’ occupations.

#### Economic evaluation

A full economic evaluation was not conducted in any of the studies meeting our inclusion criteria. Nevertheless, five studies measured the impact of services on healthcare staffing, appointments and prescribing,^47 50–52 57^ three studies calculated financial returns to participants^50 56 57^ and one study reported the cost of delivering the service^47^ (table 4).

**Table 4:** Effectiveness of interventions on primary and secondary outcomes

Study (year)	Quality rating	Effect on mental health outcomes
*Welfare and advice services located in healthcare settings*		
Abbott and Hobby (2000)^53^	Low	For participants with increased income after 6 months (n=48), improved mean scores for some SF-36 domains: vitality (pre: 20.8, post: 28.5, t=3.3, p=0.002), emotional role functioning (pre: 36.8, post: 51.4, t=2.2, p=0.037) and mental health (pre: 45.9, post: 53.1, t=2.9, p=0.005), but not social functioning (pre: 29.4, post: 32.0, p>0.05). No improvements observed at 6 months in participants without increased income (n=20), or in either group after 12 months.
Caiels and Thurston (2005)^56^	Low	For those participants who complete preintervention and postintervention questionnaires (n=81), no change in SF-12 score (pre: mean=34.1, post: mean=35.6, p=0.335). Over the 12-month period, £356 754 gained on behalf of clients.
Abbott *et al* (2006)^54^	Low	At 6-month follow-up, no improvements in SF-36 domains (vitality, social functioning, emotional role and mental health) among those who saw an increase in income following participation (n=160) compared with those who did not (n=84). At 12-month follow-up, improvements observed in emotional role (adjusted mean difference: 16.37 (2.72–30.01), p=0.02) and mental health (adjusted mean difference: 6.85 (0.72–12.98), p=0.03) for participants with increased income (n=134), compared with those who did not (n=50). No change in vitality and social functioning scores between the two groups.
Harris (2013)^57^	Low	Improvements in mean emotional well-being scores (preadvice: 47.8, postadvice: 61.3, t=3.3, p=0.001) and role limitation due to emotional problems (preadvice: 35.1, postadvice: 62.2, t=3.2, p=0.002) observed at case closure (n=65). 35% of participants achieved financial gains during the programme (mean gain £4686 per benefiting client). 17 weeks of staff time saved over the 3-year study period, resulting in an annual savings of £8700.
Ryan *et al* (2012)^55^	Low	Compared with baseline, participants (n=67) reported decreased stress (mean difference: 8.1, p<0.001) and increased well-being (mean difference: 1.8, p<0.001) after participation in the programme.
Krska *et al* (2013)^52^	Low	In the 6-month period following participation, no changes observed in mean number of primary care mental health appointments, mental health referrals or antidepressant prescriptions per patient. However, a decrease was observed in mean prescriptions for hypnotics/anxiolytics per patient (−0.16, p<0.05).
Woodhead *et al* (2017)^50^	Moderate	Overall, no evidence of effect on probable common mental disorder or well-being. However, relative to controls, probable common mental disorder reduced for female (rOR=0.37 (95% CI 0.20 to 0.70)) and Black advice recipients (rOR=0.09 (95% CI 0.03 to 0.28)). Well-being increased for participants who received a positive outcome from the advice service (β coefficient=1.29, 95% CI 0.25 to 2.32, p=0.015). No evidence that the intervention impacted 3-month, self-reported consultation frequency. £2689 average financial gain per participant over the study period. £15 income gain per £1 provided by the funder.
*Link worker social prescribing*		
Grant *et al* (2000)^47^	Moderate	After adjustment for baseline scores, participants showed greater reductions in anxiety scores (−1.9, 95% CI −3.0 to −0.7, p=0.002), but not depression scores (−0.9, 95% CI −1.9 to 0.2, p=0.116). Participants showed greater improvement in functional health components relating to pain (−0.5, 95% CI −0.8 to −0.1, p=0.005), emotional feelings (−0.5, 95% CI −0.8 to −0.2, p=0.003), ability to carry out everyday activities (−0.5, 95% CI −0.8 to −0.2, p=0.001) and feelings about general health (−0.4, 95% CI −0.7 to −0.1, p=0.003). No difference was observed between groups in social support.
Grayer *et a*l (2008)^51^	Low	Compared with baseline, fewer participants experiencing psychological distress based on both the GHQ-12 (pre: 82.6%, post: 52.2%, difference: 30.4% (16.9–43.9)) and the CORE-OM (pre: 85.1%, post: 67.6, difference: 17.5% (7.4%–27.7%)). The proportion of patients prescribed psychotropic medication declined (pre: 34.7, post: 18.8%, difference: 15.8% (6.0–25.6)), while the proportion receiving onward mental health referrals increased (pre: 7.9%, post: 19.8%, difference: 11.9% (1.9–21.9)).
*Telephone debt advice services*		
Jinhee Kim *et al* (2013)^58^	Low	At 18-month follow-up, small improvements in health scores were observed in participants (n=70) (pre: 10.60, post: 10.98, t=2.62, p<0.05) but not in controls (n=100) (pre: 10.68, post: 10.60, t=0.29, p>0.05).
Pleasence and Balmer (2007)^48^	Low	Trial stopped early due to attrition. However, after 20 weeks, no changes were observed in anxiety or general health score for either the intervention group (n=119) or the control group (n=115).
*Food insecurity interventions*		
Roncarolo *et al* (2016)^61^	Low	Improved mental health scores were observed for participants in traditional food security interventions (pre: 58.1, post: 63.9, adjusted β coefficient: 5.3 (3.1–7.4)), while no changes were observed in participants of alternative interventions (pre: 66.1, post: 71.1, adjusted β coefficient: 4.2 (−1.3 to 9.7)).
*Active labour market programmes*		
Rose (2019)^59^	Low	Labour programmes that most closely replicate employment (wage subsidies and subsidised self-employment) had the largest effect on improving the well-being of participants. When results were disaggregated by sex, no differences in well-being were observed participating in the ALMP schemes, compared with non-participants.
Saloniemi *et al* (2014)^60^	Low	Overall, no change in psychological distress, sense of coherence or stress at the end of the training course, compared with baseline. However, improvements in all three measures were seen among participants with a tertiary education and those who were previously employed in a ‘white-collar’ occupation.
Vinokur *et al* (2000)^49^	Moderate	At 2-year follow-up, compared with controls, participants had significantly higher role functioning and lower depressive symptoms, and were less likely to have experienced a probable major depressive episode.

CORE-OM, Core Outcome Measurement tools; GHQ-12, General Health Questionnaire; SF-12, 12-item Short Form Survey; SF-36, 36-item Short Form Survey.

Interviews with healthcare workers revealed a perception that colocated welfare and advice services reduced staff workloads^57^ and the number of GP appointments, mental health referrals and prescriptions.^52^ However, changes to the frequency of GP consultations were not confirmed using either retrospective analysis of medical records^52^ or patient self-report.^50^ Colocated welfare advice services produced substantial financial gains for participants due to increased welfare payments and reduced debt.^50 56 57^ A return on investment analysis of one service showed a total financial gain to participants of £15 for every £1 spent by the service provider after 8 months.^50^ A link worker service found that participants received more mental health prescriptions and fewer mental health referrals.^47^ However, the intervention arm accrued a higher mean cost per participant compared with controls after the cost of delivering the service was taken into account.

## Discussion

This review identified five categories of community interventions for working-age adults during periods of financial adversity. None of the included studies received a high-quality assessment and we find insufficient evidence to conclude that any one of these diverse categories of interventions is effective for improving mental health and well-being. Further, none of the studies identified conducted a full economic evaluation and there is limited evidence of an impact on costs to the health system.

Three studies without a high risk of bias showed some evidence of effectiveness.^47 49 50^ Participation in group job skills training reduced the symptoms of depression and enhanced emotional functioning,^49^ which aligns with the wider literature confirming the effectiveness of similar job search programmes on the mental health of the long-term unemployed.^63 64^ There was some evidence that the provision of legal and welfare advice in primary care^50^ and referral to community sector programmes^47^ improved outcomes, at least for some subpopulations. The remaining studies received low-quality assessment ratings, primarily due to self-selected samples,^53 55 57 58^ uncontrolled designs^51–57 60^ and poor study retention.^48 53 54 58 61^ Four studies used primary care and mental health service utilisation as outcome measures.^47 50–52^ However, it is unclear whether these outcomes indicated improved access to services or quality of care, or declining mental health.

This overall lack of evidence mirrors the findings of reviews examining the effect on health and social outcomes of colocated advice services^65^ and social prescribing,^66^ and interventions to reduce the impact of poverty and inequality,^67^ and unemployment and economic hardship.^41^ Studies identified in our review described the challenges of conducting trials in populations experiencing financial uncertainty^48^ and within primary care and voluntary sector settings^47^ highlighting the difficulties of measuring the impact of community interventions using traditional effectiveness studies.^67 68^

The studies we identified provide useful insights for intervening on health inequalities, which is particularly important in light of emerging evidence that the COVID-19 pandemic has exacerbated existing patterns of inequalities.^69–71^ Since the introduction of measures to contain the pandemic, those living in deprived communities and low-income households have been more likely to experience both financial adversity (employment sector shutdowns, reduced income and job losses)^69 70^ and declining mental health.^71^ Most interventions we identified aimed to reach deprived communities, either through targeted delivery in deprived neighbourhoods^50 52 53 56^ or through eligibility based on income or employment status.^48 49 55 59 60^ As might be expected, there was some evidence that mental health outcomes improved only for those participants who saw improvements in their financial circumstances.^49 50 53 54 61^ This indicates that the social determinants of health are amenable to change through focused community interventions and that reducing financial uncertainty could be an actionable pathway towards improved mental health.

Colocated welfare and advice services produced substantial financial gains for participants.^50 56 57^ This underscores the capacity for these programmes to correct the historical underclaiming of welfare entitlements observed in many deprived communities,^65^ which has been highlighted as a persistent problem during the implementation of COVID-19 employment protection policies.^72^ Nevertheless, evidence from vocational training programmes showing psychological distress reduced only among participants previously employed in salaried professions or with a tertiary education warns of the potential for community interventions to widen health inequalities.^60^

There is some evidence that integrating community services into healthcare settings can improve mental health,^47 50^ which is important within a policy context that increasingly emphasises third sector organisations’ role in providing care.^73^ Nevertheless, all 13 studies that adequately reported sample characteristics recruited more female participants than males (range 52%–75%). As more than half of our studies recruited participants from GPs, this may in part reflect lower primary care consultation rates observed in men.^74 75^ Therefore, variation in service utilisation and quality of care by gender, age, economic deprivation and language and ethnicity^76–78^ should be considered when implementing programmes in these settings.

### Limitations

A focus on universal and selective prevention during the acute stages of financial adversity means we have systematically excluded studies evaluating community interventions on specific subpopulations with recognised pre-existing challenges, such as the long-term unemployed and homeless, and people with severe mental illness. Similarly, we excluded a wider body of literature examining the effectiveness of interventions that seek to address chronic financial hardship and neighbourhood-level poverty.^79 80^ In general, the interventions we identified were not mental health interventions. Instead, their primary aim was to improve the material circumstances of participants. Therefore, we will have excluded studies evaluating the impact of these, and other promising interventions, on non-mental health outcomes, particularly those operating outside of healthcare settings where mental health outcomes are not routinely collected. We included only studies published since 2000 in order to identify evidence most relevant to contemporary service configuration and contexts; however, this may have introduced some bias. Reviewing studies from across high-income countries with divergent welfare provision models means the effect of an intervention observed in one country is unlikely to be generalisable to another.^81^ Finally, excluding qualitative research and process evaluations further limits our findings. For many studies, limited information could be extracted regarding the intervention setting, the procedures and resources involved and any underpinning theory of change. A better understanding of the mechanisms through which these interventions act and the pathways towards improved mental health is required.^82^

## Conclusion

The evidence base for community interventions delivered to individuals experiencing personal or household financial adversity was marked by the presence of small, uncontrolled studies drawn from self-selected samples. However, our review highlights that community interventions can successfully reduce financial uncertainty, which is increasingly important given the deteriorating economic situation caused by the COVID-19 pandemic.

**What is already known on this subject**

- Episodes of financial adversity, such as recent job loss, precarious employment, personal debt and housing and food insecurity, represent a crucial period for the protection and promotion of mental health in working-age adults.
- Community interventions are one means through which to address these social determinants of mental health.
- A stronger evidence base regarding the effectiveness of community interventions delivered to the general population during periods of financial insecurity is required.

**What this study adds**

- The evidence base for effective and cost-effective community interventions is marked by the presence of small, uncontrolled studies including potentially non-representative samples.
- Nevertheless, job search programmes, welfare and advice services located in healthcare settings, and link worker referral services show some evidence of effectiveness, at least for some subpopulations.
- The social determinants of health are amenable to change through focused community interventions and mitigating the impact of financial adversities could be an actionable pathway towards improved mental health.
