# Supplementary material for: Effectiveness of community interventions for protecting and promoting the mental health of working-age adults experiencing financial uncertainty: a systematic review
Source: J Epidemiol Community Health. 2021 Apr 30;75(7):665–73. doi: 10.1136/jech-2020-215574 (PMC8223661; doi:10.1136/jech-2020-215574)
Supplement: Supplementary data [file jech-2020-215574supp001.pdf]

Supplemental material: Medline search terms

1. (psycho?social adj (problem* or adversit* or cris?s))
2. unemployment/ or Personnel Downsizing/
3. ((precarious* or insecur* or secur* or contingent or marginal* or minimal* or terminat* or loss) adj3 (employ* or work* or job*))
4. (jobless* or unemploy* or redundan*)
5. poverty/ or vulnerable population/
6. ((financ* or economic* or housing or rent* or mortgage or food) adj4 (secur* or insecur* or hardship* or difficult* or worry or worries or strain* or anxiety or stress* or distress))
7. (bankrupt* or debt)
8. bankruptcy/
9. (risk adj3 homeless*)
10. (eviction or evicted or repossess*)
11. 1 or 2 or 3 or 4 or 5 or 6 or 7 or 8 or 9 or 10

12. government programs/ or public policy/ or social welfare/ or social work/ or community networks/
13. primary prevention/
14. (mental health adj3 (prevention or promotion))
15. ((financial or income or debt) adj3 (advice or support or assistance or service or counse?ling))
16. ((employment or job) adj3 (advice or support or assistance or service* or counse?ling))
17. ((welfare or benefits or legal) adj3 (advice or support or assistance or service* or entitlement or rights or advoca*))
18. ((housing or tenan*) adj3 (advice or support or assistance or service*))
19. (food bank or community food program* or community kitchen)
20. ((community or peer or citizen*) adj3 (advice or support or assistance))
21. ((advocacy or support or employment or job* or welfare or housing or wellbeing or well-being or food insecurity) adj (intervention* or program* or scheme))
22. (community intervention* or community-based intervention* or community scheme or community program* or social intervention)
23. (community referral* or referral scheme or referral* facilitat*)
24. (information adj (referral or advice or service*))
25. 12 or 13 or 14 or 15 or 16 or 17 or 18 or 19 or 20 or 21 or 22 or 23 or 24

26. mental health/ or Stress, Psychological/ or anxiety/ or depression/ or "Quality of Life"/ or Personal Satisfaction/ or Emotional Adjustment/ or Resilience, Psychological/
27. ((psycho* or emotional* or mental) adj (distress* or stress or health or well?being))
28. (depressi* or anxiety or hopeless*)
29. (resilience or happiness or self efficacy or self esteem)
30. 26 or 27 or 28 or 29
31. 11 and 25 and 30
